# Letter to the Editor

**DOI:** 10.52054/FVVO.14.4.052

**Published:** 2023-01-27

**Authors:** P.R. Koninckx, A Ussia, S Gordts

**Affiliations:** Latifa Hospital, Dpt OBGYN, Dubai, United Arab Emirates; Prof Emeritus OBGYN,KU Leuven, Belgium; University of Oxford and Hon Consultant, UK; University Cattolica, Roma, Italy; Moscow State University, Russia; Consultant Università Cattolica, Dpt OBGYN, Roma, Italy; Life Expert Centre, Leuven, Belgium

Sir,

Your article ‘Endometrial preservation during resection of type II and type III submucosal fibroids’ (Vorona and Saridogan, 2022) and the discussion on the healing of the endometrium, was read with interest. However, hysteroscopic resection of type II or type III fibroids will also incise or resect the junctional zone (JZ). Checking Pubmed for (hysteroscopy OR myoma) AND (JZ or junctional zone) not a single article was found discussing the trauma and the healing of the JZ following hysteroscopic myomectomy. Only one article studied the healing of the myometrium after abdominal myomectomy without mentioning the JZ (Tsuji et al., 2006). Abdominal myomectomy of large myomas decreases uterine contractility, which was suggested to improve fertility (Yoshino et al., 2012).

The JZ, or archimetra, is different from the myometrium as seen on MRI and ultrasound imaging. The JZ, Müllerian in origin, is responsible for peristaltic concentric uterine contractions (Leyendecker et al., 2022). The JZ plays a major role during placentation (Koninckx et al., 2018) and in the development of adenomyosis. High intrauterine pressures causing severe dysmenorrhoea were explained by excessive contractility of the stratum vasculare of the neometra (Leyendecker et al., 2022).

Considering the importance of the JZ in reproduction and the intimate relationship with the endometrium and uterine contractility, we were wondering whether you could discuss the repair of the junctional zone following hysteroscopic myomectomy and the importance of reducing the trauma. Besides the scientific interest, a hysteroscopic linear incision during hysteroscopy could eventually be considered as a treatment of dysmenorrhoea and eventually as a prevention of endometriosis.
